# Meta‐omics assisted microbial gene and strain resources mining in contaminant environment

**DOI:** 10.1002/elsc.202300207

**Published:** 2023-08-18

**Authors:** Yiqun Huang, Haiyang Hu, Tingting Zhang, Weiwei Wang, Wenzhao Liu, Hongzhi Tang

**Affiliations:** ^1^ State Key Laboratory of Microbial Metabolism and School of Life Sciences & Biotechnology Shanghai Jiao Tong University Shanghai People's Republic of China; ^2^ China Tobacco Henan Industrial Co. Ltd. Zhengzhou People's Republic of China

**Keywords:** gene mining, meta‐omics approaches, pollutants, yet‐to‐culture strains

## Abstract

Human activities have led to the release of various environmental pollutants, triggering ecological challenges. In situ, microbial communities in these contaminated environments are usually assumed to possess the potential capacity of pollutant degradation. However, the majority of genes and microorganisms in these environments remain uncharacterized and uncultured. The advent of meta‐omics provided culture‐independent solutions for exploring the functional genes and microorganisms within complex microbial communities. In this review, we highlight the applications and methodologies of meta‐omics in uncovering of genes and microbes from contaminated environments. These findings may assist in future bioremediation research.

**Abbreviations**
AMD: Acid mine drainage
BLAST: Basic local alignment search tool
FACS: Fluorescence activated cell sorting
HMM: Hidden Markov model
IMG: Integrated microbial genome
LIVEST: Laser induced visualized ejection separation technology
MAGs: Metagenome‐assembled genomes
MALDI‐TOF: Microorganisms with matrix‐assisted laser desorption ionization time‐of‐flight
ORFs: Open reading frames
PCBs: Polychlorinated biphenyls
PET: Polyethylene terephthalate
PLA: Polylactic acid

## 1 INTRODUCTION

Industrial production and agriculture contribute a large quantity of environmental pollutants. The increase of pollution (e.g., microplastics, polycyclic aromatic hydrocarbons, organohalides, pesticide, and heavy metals) has become a crucial environmental issue [1, 2, 3, 4, 5]. These pollutants are persistent and difficult to remove due to their high stability and various of toxic effects exerted on the human body and ecosystem [6]. Several physical and chemical methods (e.g., adsorption, filtration, incineration, and redox reactions) have been utilized for pollutant remediation [6]. Additionally, microbial‐based bioremediation technologies (e.g., activated sludge, biofilm, and bioleaching) are often considered to be an efficient and environmentally safe alternative for pollutant removal [7, 8, 9]. In earlier studies, many microorganisms (e.g., *Alcaligenes*, *Cellulosimicrobium*, *Sphingobium*, and *Flavobacterium*) and enzymes (e.g., oxidoreductases, hydrolases, and amidohydrolases) have been applied in environmental bioremediation and industrial production [6, 10].

Microbial communities that exist in long‐term contaminated environments (e.g., wastewater, refinery waste, and acid mine drainage [AMD]) are often assumed to possess the capacity of pollutant tolerance or degradation [11, 12]. The presence of these degrading genes and microorganisms in microbial communities is vital to enhancing the efficiency of existing bioremediation methods and the exploration of novel treatment approaches and degradation pathways of pollutants. However, the “great plate count anomaly” phenomenon indicates that only a small proportion of microorganisms are capable of growth or isolation using current techniques [13]. The unclear and complex growth conditions of microorganisms are a barrier to their successful culturing and isolation. Potential reasons include the demand for substrates, suitable environmental conditions (e.g., temperature, pH, and salinity), resuscitation of dormancy, and symbiotic interdependencies [14].

The utilization of meta‐omics approaches (e.g., metagenomics, metatranscriptomics, and metaproteomics) can obtain the different molecular information (e.g., gene, RNA, and protein) of microbial communities in contaminated environment. This information aids researchers to in their analysis or prediction of potential metabolic characteristics of in situ microbial communities [15, 16, 17]. Metagenomics is a method utilized to obtain all genomes of entire microbial communities to investigate potential DNA information in samples. The assembly and annotation of DNA can be utilized to evaluate species composition and gene functions present in microbial communities [15]. Metatranscriptomics is able to measure the dynamic changes of mRNA in microbial communities and the expression level of different genes [16]. Metaproteomics measures the abundances of proteins in microbial communities across different environments (e.g., activated sludge, and AMD) through protein sequence databases [17]. This approach can be applied to discover how proteins respond to pollutants in contaminated environments. To date, many meta‐omics datasets from diverse environments have been sequenced and released in public databases. Researchers can perform functional annotation or establish sequence libraries for activity screening based on the available public or private meta‐omics datasets to uncover novel enzymes [11].

**FIGURE 1:**
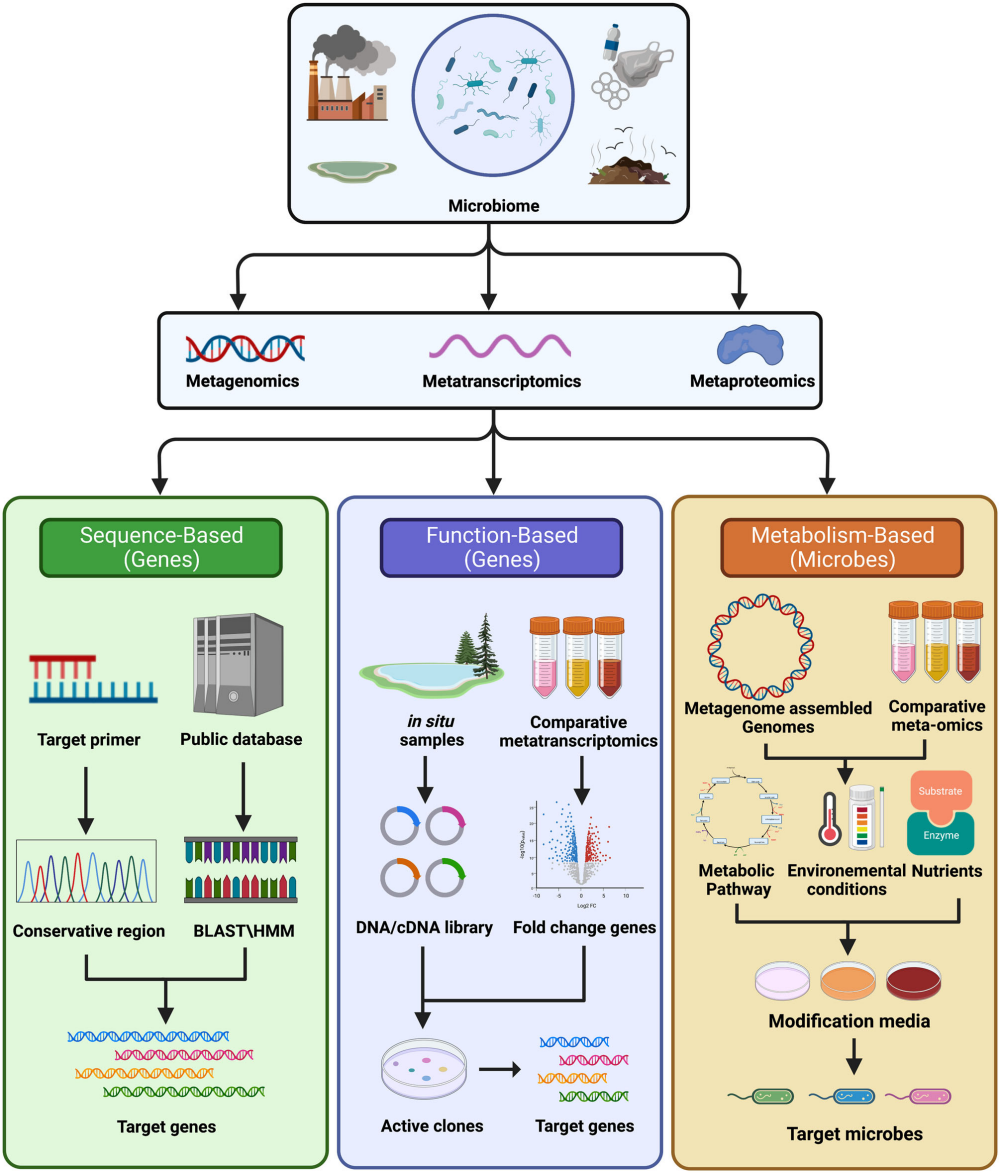
The workflow of meta‐omics approaches to explore and mine the microbial genes and strains in complex environmental microbiomes (Created with BioRender).

Metagenome‐assembled genomes (MAGs) are able to be partially reconstructed from microbiome metagenomic reads through metagenomic binning [18]. The physiology and ecological requirements of the microbial individual genome can be analyzed by MAG, lending guidance for the enrichment or isolation of target microorganisms and help obtain yet‐to‐culture microorganisms [19]. In this review article, we present various research studies related to (1) Meta‐omics‐assisted discovery of pollutant metabolic genes; (2) Meta‐omics guided isolation of functional strains of environmental microbiota. These cases demonstrate how to use different methods (e.g., sequence‐based strategy, function‐based strategy, and metabolism‐based strategy) to design a workflow to obtain the target enzymes and strains through meta‐omics datasets. This will aid future research on bioremediation of contaminated environments. (Figure 1)

## 2 META‐OMICS ASSISTED THE DISCOVERY OF POLLUTANT RELATIVE GENES

### 2.1 Sequence‐based metagenomics

In early studies, target‐specific primers were designed based on the conserved sequences of functional genes or operons. The metagenomic DNA of environmental samples were extracted, and PCR amplification was performed to obtain the potential target DNA fragments, enabling the function of sequences to be validated [20, 21, 22]. Obtaining the flanking regions of the target fragment is crucial to obtain the complete gene via PCR. Morimoto employed PCR‐denaturing gradient gel electrophoresis to obtain target fragments that may contain benzoate dioxygenase (*benA*) and chlorocatechol dioxygenase (*tfdC*) in 3‐chlorobenzoate‐contaminated soil samples. Subsequently, metagenome walking was employed to successfully obtain the full‐length gene sequences of *benA* and *tfdC* [20]. Isotopically labeled pollutants (e.g., ^13^C, and ^15^N) can be utilized by microorganisms as carbon or nitrogen sources. Therefore, stable isotope probing provides the possibility of obtaining the labeled DNA of pollutant‐degrading microorganisms. In a study, Sul added ^13^C‐biphenyl to the polychlorinated biphenyls (PCBs) contaminated River Raisin sediment and conducted a long‐term cultivation of indigenous microbial communities. The ^13^C‐DNA was separated from the post‐cultivation DNA samples using isopycnic centrifugation. Target aromatic‐ring‐hydroxylating dioxygenase gene primers were used for cosmid library construction and screening with labeled DNA via PCR. Biphenyl dioxygenase subunit (*bphAE*) genes were found in a cosmid clone, which showed activity towards biphenyl and PCBs in *Escherichia coli* BL21 [21].

Similar sequences may arise through evolution from a common ancestry. Many catalytic active sites in enzymes are highly conserved among different genes or proteins. BLAST (Basic local alignment search tool), which is based on sequence similarity, and HMM (Hidden Markov model), which is based on functional domains are important tools for gene or protein annotation in meta‐omics datasets [23, 24]. With the increasingly updated of many large comprehensive databases (e.g., KEGG, eggNOG, and Uniprot), as well as the release of small specific functional gene databases (e.g., VFDB, BacMet, and NCcyDB), and the development of pollutant biodegradation resources databases (e.g., mibPOPdb, OxDBase, and EAWAG‐BBD) [25, 26, 27, 28, 29, 30, 31, 32, 33]. Function prediction of metagenomic genes has been accelerated and can be used to evaluate the metabolic potential of microbial communities.

Based on the metagenomic data from a continuous trichloroethylene dechlorinating microbial community, Brisson used Integrated Microbial Genome (IMG) system for gene prediction and annotation. The study identified putative reductive dehalogenases from the metagenomic database [34]. Due to differences in the deposition and classification rules of public databases, a sequence may yield diverse annotation results across databases. Therefore, researchers need to conduct multi‐databases comparisons and analyses to obtain more comprehensive outputs. For example, Danso developed an HMM to search the existing public databases of metagenomic and genomic data (e.g., UniProtKB, RefSeq, and IMG) to identify new polyethylene terephthalate (PET) hydrolases [35]. The researchers developed a model based on nine validated PET hydrolase sequences. Visual analysis of the HMM shows the important motifs which are associated with enzyme thermostability, substrate binding, and catalytic activity. 504 putative PET hydrolase sequences were retrieved from the database throughout the search. 13 sequences were manually selected based on sequence similarity and motif for further functional validation, four candidate genes present the activity in heterologous expression experiment [35]. This sequence‐based metagenomic strategy has demonstrated the ability to uncover new pollutant‐degrading enzymes from microbial communities.

Enzymes with similar catalytic mechanisms may be identified using sequence similarity and conservation. However, many unknown sequences in metagenomic data that cannot be correctly annotated using current databases, and many pollutant metabolic pathways and mechanisms are uncharacterized or not investigated.

### 2.2 Function‐based metagenomics

Function‐based metagenomics divides the original metagenomic DNA into fragments via restriction enzymes digestion or mechanical forces. The DNA fragments are then transferred into vectors to establish a metagenomic library. Several genetically well‐characterized and manipulation‐friendly expression hosts (e.g., *E. coli*) were selected for heterologous expression of the genes present in the metagenomic library. Various high‐throughput strategies have been designed to demonstrate the activity of genes or gene clusters [11, 36]. Many novel enzymes have been discovered and employed in the bioremediation of different pollutants (e.g., aromatic compounds, plastic, and pesticide), which are outlined in Table 1.

**TABLE 1:** The mining cases of microbial genes and strains via meta‐omics methods.

Gene/Strain	Method	Pollutant	Source	Reference
PET hydrolases	MG (seq)	Plastic	Public database	[35]
Reductive dehalogenases		Organohalide	Sediment	[34]
Naphthalene degradation gene		Aromatic compounds	Groundwater	[22]
Biphenyl dioxygenase			Sediment	[21]
Benzoate 1,2‐dioxygenase			Soil	[20]
Aromatic dioxygenase			Soil	[91]
Extradiol dioxygenase	MG (fun)	Aromatic compounds	Activated sludge	[40]
Phenol hydroxylase			Sludge	[92]
PLA depolymerase		Plastic	Compost	[45]
Cutinase			Compost	[42]
Esterases			Compost	[43]
Monooxygenase			Soil	[93]
2,4‐dichlorophenol hydroxylase		Herbicide	Soil	[51]
Pyrethroid‐hydrolyzing esterase		Pesticide	Soil	[49]
Esterases			Rumen	[50]
Metal resistance gene		Heavy Metal	Rhizosphere	[53]
Cr(VI) remediation gene	MT	Heavy Metal	Soil	[57]
Metal resistance gene			Soil	[5]
Metal resistance gene			Soil	[59]
Metal resistance gene			Soil	[60]
Metal resistance gene			Soil	[94]
Metal resistance gene			Soil	[58]
*Leptospirillum ferrodiazotrophum*	MG	Heavy Metal	Acid mine drainage	[75]
*Paraclostridium* sp. strain EML	MG, MT, MP	Arsenic	Soil	[77]
*Microbacterium* sp. J1‐1	MD, MG	Organohalide	Activate sludge	[78]

a) MG (seq): Sequence‐based metagenomic analysis; MG (fun): Function‐based metagenomic analysis; MG: Metagenomic analysis; MT: Metatranscriptomic analysis; MP: Metaproteomic analysis; MD: Microbial diversity analysis.

#### 2.2.1 Aromatic compounds

Aromatic compounds and polycyclic aromatic hydrocarbons (PAHs) are common pollutants in petroleum industries or municipal wastes [37]. These compounds may bioaccumulate in humans and possess genotoxic, carcinogenic, mutagenic, and teratogenic properties, making them a significant risk of environment [38].

Extradiol dioxygenase is a key enzyme responsible for the catalysis of aromatic compounds. It can cleave catecholic substrates to form linearized products for subsequent central metabolism [39]. A study by Suenag used activated sludge from a coke plant wastewater as samples and established a metagenomic library in fosmids based on the length range (10‐30 kb) of the aromatic compounds degradation gene clusters. Extradiol dioxygenase can catalyze the production of a yellow product from catechol, which can used to quickly identify positive clones based on enzyme activity. The researchers identified 43 extradiol dioxygenase genes from 38 positive clones, and 25 of genes belonged to 4 novel subfamilies [40].

#### 2.2.2 Plastic

PET is a common plastic material with widespread applications. PET has low hydrophilicity and high physical stability, which make it become an environmental pollution issue due to its difficulty in degrading. Cutinase has been reported to degrade various polymers (e.g., PET, polyacrylonitrile, and polyamide) [41]. A metagenomic library was constructed based on plant leaf‐branch compost to screen for cutinase homologs. Clones possessing cutinase activity were able to degrade tributyrin and form a halo on the culture medium. A study conducted by Sulaiman identified 19 clones with a halo on tributyrin agar plates which verified the PET degradation activity of a novel cutinase homolog [42]. Kang also used tributyrin agar plates to identify for a novel esterase gene *estCS2* from a compost metagenome. This enzyme was able to maintain activity at high temperatures (60°C) and a broad pH range (pH 6–10) for the degradation of polyurethanes [43]. Polylactic acid (PLA) is known as an alternative material to ordinary plastics, which was found to degradation in compost [44]. Mayumi extracted metagenomic DNA from surface samples of PLA disks in compost, and emulsified‐PLA agar plates were used for activity screening. Researchers obtained a total of seven positive clones and successfully purified three clones, while the purified proteins were able to degrade PLA and other emulsified polyesters [45].

#### 2.2.3 Pesticides and herbicides

Several pesticides (e.g., pyrethroids, and chlorpyrifos) and herbicides are widely used for pest and weed control in agricultural production and household living. Residues from pesticides and herbicides found in fruits, vegetables, and farmland result in health risks to humans. Pyrethroids can induce endocrine disruption and carcinogenic effects in humans, while 2,4‐dichlorophenoxyacetic acid may result in abdominal pain, hypotension, and myotonia [46, 47]. In a recent project, Li extracted and constructed a metagenomic library based on vegetable soil and identified 6 positive clones with esterase activity that produced blue products via X‐caprylate. Only 1 new pyrethroid‐hydrolyzing gene (*pye*) was successfully heterologously expressed in *E. coli* BL21 and demonstrated a broad substrate catalytic activity (e.g., cypermethrin, permethrin, fenvalerate, deltamethrin, and malathion) [48]. A study by Fan employed a similar approach to obtain a family V esterase from Turban Basin soil, which demonstrated the ability to degrade various substrate [49]. Many pesticide‐degrading enzymes (e.g., esterase, and 2,4‐dichlorophenol hydroxylase) have been discovered from metagenomic libraries and can be investigate in future bioremediation research [50, 51].

#### 2.2.4 Environmental stress resistance

Various environmental conditions (e.g., pH, temperature, and salinity) may fluctuate due to seasonal variations or geographical distribution. Genes that maintain microbial or enzyme degradation activity under different environmental conditions are critical for bioremediation.

Guazzaroni constructed six metagenomic libraries from the extreme acidic and heavy metal‐enriched environment (Tinto River) and used acid shock assays to select for acid‐resistant clones via calculating the survival rate after acid treatment. In total, 15 putative acid resistance‐related open reading frames (ORFs) were identified. To deal with the potential host‐dependent issues of gene expression, the function of 9 ORFs was validated using three microbial hosts (*E. coli*, *Pseudomonas putida*, and *Bacillus subtilis*), 4 ORFs (*Ard2*, *Ard3*, *HU*, and *LexA*) were shown to enhance acid resistance in multiple hosts [52]. Mirete primarily focused on the identification of heavy metal resistance genes in the Tinto River's heavy metal‐contaminated environment [53]. Researchers collected samples from the rhizosphere of plants growing along the riverbank and selected metagenomic library clones using culture media containing lethal concentrations of Ni. A total of 13 nickel resistance‐positive clones were obtained, six genes were first reported as nickel resistance [53].

Environmental factors associated with various contaminated samples may impact the adaptive evolution of enzymes. A study by Tchigvintsev used crude oil to enrich and culture in oil‐ and PAHs‐contaminated seawater samples (cold environment). Five esterases were obtained from the metagenomic library and purified from *E. coli*. These esterases retained the capacity to degrade pollutants under low temperatures (5°C) [54]. In contrast, Fan identified esterases capable of maintaining activity at high temperatures (55°C) from Urban Basin soil samples (hot environment) [49].

The high standard of DNA fragments and expression hosts are the important limitations to function‐based metagenomics. For example, the completeness of genes or gene clusters found in DNA sequence fragments should be sufficient to show functional phenotypes. Furthermore, several functional genes may become inactive without transcriptional regulators or activators, even if their own genes are complete. Heterologous genes should be expressed in the host microorganisms, and the protein sequences should be folded and modified normally in the cells. Moreover, the hosts should be able to tolerate the transcriptional interference and toxicity caused by heterologous gene expression. The limitation of DNA can be partially alleviated by the insertion of large fragments into vectors, but the effectiveness of this method is unstable. The problem of hosts can be improved by testing multiple host microorganisms (e.g., *E. coli, Agrobacterium tumefaciens*, and *P. putida*) or using in vitro reconstituted transcriptional systems to increase the successful possibility of heterologous expression [11, 36].

### 2.3 Metatranscriptomics

Transcription levels of specific genes from microbial communities may be altered when microorganisms are exposed to exogenous pollutants [55]. Researchers can identify the activity of genes under specific conditions, allowing for the analysis of metabolism within microbial communities in contaminated environments via metatranscriptomics [56].

Research by Pei observed a significantly upregulation in the expression of the representative Cr(VI) remediation genes (*chrA*, and *yieF*) after 30 min of Cr(VI) treatment in microbial samples via qPCR [57]. Researchers conducted comparative metatranscriptomics and metagenomics on heavy metal‐contaminated soil in the presence and absence of Cr(VI) treatment. In Cr(VI)‐treated group, 77 upregulated genes were found with no annotations in the KEGG and GO databases. Six genes had complete ORFs in the metagenomic library, which were transferred into *E. coli* to verify their phenotypic functions. All engineered strains had enhanced chromium resistance or Cr(VI) reduction capabilities compared to the negative control [57].

Similar to function‐based metagenomic approaches, the cDNA of metatranscriptomics can be employed to construct various clones for the discovery of novel genes. In one study, Mukherjee, based on a cDNA library from metal‐contaminated soil, isolated copper tolerance genes (*PLCc38*) in metal‐sensitive yeast mutants [58]. Lehembre and Thakur also separately screened resistant genes from soil metatranscriptomes with various heavy metals (e.g., Cu, Zn, Co, and Cr) [5, 59, 60].

These studies indicate that metatranscriptomics is useful in facilitating the discovery of novel genes. The limitations of metatranscriptomics include RNA instability, ineffective cell lysis, presence of RNA enzymes, and RNA adsorption will reduce RNA extraction efficiency [16, 61].

### 2.4 Metaproteomics

Metaproteomics does not construct DNA or cDNA libraries for functional activity screening as in metagenomics and metatranscriptomics. The reference sequence database directly affects annotation and classification of proteins. The database can be obtained from metagenomic data of similar samples in public databases or from corresponding metagenomic or metatranscriptomic data from the same sample [17]. Metaproteomics can attribute metabolic functions across different environments to specific microbes within diverse microbial communities, which is useful for identifying pollutant biodegradation processes and investigating microbial systems ecology [62, 63]. Benndorf conducted a metaproteomic analysis of 2,4‐dichlorophenoxy acetic acid‐contaminated soil and identified presence of chlorobenzene dioxygenase and chlorocatechol 1,2‐dioxygenase, with at least 2 species involved in the biodegradation of chlorobenzene [62]. Compared to metagenomics, metatranscriptomics, and comparative proteomics centered on pure cultures, fewer studies employ metaproteomics to directly explore novel pollutant degradation or resistance genes within microbial communities [63, 64].

## 3 META‐OMICS GUIDED ISOLATION OF ENVIRONMENTAL STRAINS

Many microorganisms remain uncultured and uncharacterized in the environment and are therefore referred to as “microbial dark matter.” They may provide important ecological contributions and represent a genetic resource for the discovery of new genes and metabolic pathways [65, 66, 67]. Many genomes of yet‐to‐culture or unculture microorganisms have been reconstructed or recovered from the environment using multi‐omics data. Numerous functional genes were predicted or selected from these microorganisms suggesting that they may possess significant potential and ecological roles in diverse environments. Therefore, isolating microorganisms from the environment is crucial for validating various ecological hypotheses derived from multi‐omics data (e.g., multi‐species interactions, evolutionary principles, and pathogenicity) [56].

Several universal approaches for the enrichment or isolation of yet‐to‐culture microorganisms have been developed (culturomics, dilution to extinction, co‐cultivation, ichip, and single‐cell sorting) [68, 69, 70, 71, 72, 73]. Culturomics relies on high‐throughput cultivation with various type of culture media, followed by species identification using matrix‐assisted laser desorption ionization time‐of‐flight (MALDI‐TOF), However, it can only identify the known microbes in microorganisms with MALDI‐TOF [68]. The dilution to extinction method dilutes the cell in each culture container to ≤1, allowing individual cell to grow without competing with other species. However, researchers have observed many mixed cultures during the cultivation process, suggesting that many microorganisms may be unable to grow independently and require co‐cultivation [69, 70]. The ichip method places diffusion chambers into the environment, allowing microorganisms to utilize unknown growth factors from the natural environment while maintaining a single‐cell state [71]. Some single‐cell sorting methods include fluorescence activated cell sorting (FACS) and laser induced visualized ejection separation technology (LIVEST). These methods have been reported to assist in the isolation of bacteria from contaminated environments (e.g., PCBs‐contaminated soil, and wastewater‐treatment plant) [72, 73]. Single cell sorting and sequencing can overcome the incompleteness of MAG to assist researchers in isolating target microorganisms.

These methods have enhanced the success rate of microbial cultivation. Furthermore, multi‐omics data can predict the unknown metabolic features of yet‐to‐culture microorganisms (e.g., substrate utilization, oxygen requirements, and antibiotics resistance), which will assist researchers in rationally designing culture protocols for the isolation of target microorganisms from the environment [19, 74]. The strategies of meta‐omics data assist the modification of culture medium (e.g., physicochemical conditions, nutrients, and antibiotics) for the target microorganism [19].

AMD is a common contaminated environment characterized by heavy metal pollution and extreme acidity. Acidophilic bacteria obtain energy through the oxidation of ferrous ions or sulfide compounds in the environment [75]. Research by Tyson reconstructed genomic fragments of five microorganisms from AMD biofilm metagenomes, including *Leptospirillum* group II and III. In contrast to *Leptospirillum* group II, which lacks nitrogen fixation genes in genome, the presence of a complete *nif* operon in the scaffold of *Leptospirillum* group III indicated its potential capability for nitrogen fixation. With the aim of specifically isolating *Leptospirillum* group III from the biofilm while excluding *Leptospirillum* group II, a customized acidic nitrogen‐free 9K medium (pH 1.2) was developed for isolation. As a result, a pure culture of *Leptospirillum ferrodiazotrophum* sp. nov was successfully isolated and identified [75]. Low‐abundance yet‐to‐culture microorganisms may have crucial metabolic activities and ecological niches in their environments. Meta‐omics data can assist in culturing of diverse microbes under laboratory conditions. In a study, Belnap employed quantitative proteomics to compare the metabolic activities of AMD microbial communities under natural and laboratory conditions. They discovered that the production of metabolic stress proteins was higher in the laboratory group, leading to a reduction in biomass accumulation. After researchers modified the 9K culture medium, the laboratory group exhibited reduced the production of metabolic stress proteins and increased the growth of microbial communities [76].

Identifying microorganisms with pollutant bioremediation‐related genes from contaminated samples using meta‐omics information, and designing isolation or cultivation strategies based on their genetic information and metabolic characteristics, will assist in obtaining pure cultures [77, 78]. Research by Viacava found that arsenic resistance (*ars*) genes were widely present in the samples (metagenomic data), but only a small part of *ars* genes were transcribed or translated (metatranscriptomic and metaproteomic data). Researchers further identified a MAG containing arsenite S‐adenosylmethionine methyltransferase (*ArsM*) via the functional gene's prediction and activity analysis of MAGs. This MAG possessed anaerobic assimilatory sulfite reductase and lacked sucrose transport or hydrolysis genes. They designed a chromogenic selective culture medium that used two metabolic features and isolated an arsenic‐methylating anaerobic microorganism (*Paraclostridium* sp. strain EML) [77]. Several organohalides (e.g., dichloropropane, and chloropropanol) are produced from industry. Huang identified 42 MAGs containing various organohalide hydrolytic enzymes in wastewater treatment plant via microbial diversity analysis and metagenomic analysis. By designing MAG screening and enrichment medium based on low‐nutrient medium (e.g., mineral salt medium), potential substrates of degradation genes, and the nitrogen and sulfur metabolic features in MAGs. Researchers isolated an organohalide dehalogenation bacterium (*Microbacterium* sp. J1‐1) which belongs to the potential biomarker and degrader class in meta‐omics analysis [78].

While the optimization of culture medium strategy can assist in strains isolation, the design of media via meta‐omics data is still unable to guarantee the isolation of target strains. Potential reasons include the incomplete MAGs, limited sequence databases to predict the physiological functions of MAGs, and some unknown or unavailable growth factors [19, 74, 79].

## 4 CONCLUDING REMARKS

Over prior decades, the number of meta‐omics projects has rapidly increased across public databases. However, single‐omics analysis in microbiome research is insufficient to fully characterize the complex physiological and biochemical phenomena of microbial communities. Integrating multi‐omics analysis allows researchers to avoid the biases of diverse omics technologies, achieve cross‐validation between omics data, and obtain more comprehensively information from microbial communities [80, 81]. Sequence‐based omics analysis is limited by the scope and quality of database sequences. Small and curated databases (e.g., CRAD, and UM‐BBD), which were developed for specific substrate degradation or tolerance will help to reduce the interference of different source sequences or incorrect annotations in large databases, improving gene annotation quality [33, 82]. Agar plate‐based positive clones have assisted in the discovery of many novel genes or proteins in function‐based omics analysis, but it has some restricted features include time‐consuming, low‐throughput, and labor‐intensive. Therefore, the development of automated, high‐throughput strategies for positive clone isolation and identification (e.g., FACS‐based screening, and microfluidics) will assist in accelerating the mining of novel pollutant degradation genes.

Optimization strategies for supplementing nutrients in culture media play an important role. Other yet‐to‐culture microorganisms isolation strategies also have been applied in non‐polluted environments (e.g., gut, marine, and deep biosphere) [83, 84, 85, 86, 87]. A study by Pope isolated strain by adding antibiotics (bacitracin) to the culture medium based on MAG antibiotic resistance. A study by Lee obtained mucin‐degrading bacteria from mouse colon using an isotope‐labeled microbial Raman‐based automated cell sorting platform. Other researchers have developed a microfluidics approach capable of isolating microbes with target genes [88, 89, 90]. Environmental pollutant‐degrading microbes may be isolated or cultivated in the future using these high‐throughput targeted strategies.

To enhance the efficiency of exploring pollution degradation genes or microbial resources, future research is needed to optimize multi‐omics technologies in the following ways: (1) modification of omics sample processing methods, and sequencing strategies; (2) development of sequences databases and biological analysis tools; (3) investigation of automated, customizable high‐throughput screening platforms; and (4) strengthening the integration between computer simulation and experimental verification. These efforts will help improve the efficiency of microbial gene and strain mining and discovery for future environmental remediation.

## CONFLICT OF INTEREST STATEMENT

The authors have declared no conflicts of interest.

## Data Availability

Data sharing not applicable to this article as no datasets were generated during the current study.
